# Fluorophotometry as a diagnostic tool for the evaluation of dry eye disease

**DOI:** 10.1186/1471-2415-6-20

**Published:** 2006-05-26

**Authors:** Magid M Fahim, Shamim Haji, Chakravarthy V Koonapareddy, Vincent C Fan, Penny A Asbell

**Affiliations:** 1 Department of Ophthalmology, Mount Sinai School of Medicine, New York, NY, USA

## Abstract

**Background:**

Dry eye disease is a common debilitating ocular disease. Current diagnostic tests used in dry eye disease are often neither sensitive nor reproducible, making it difficult to accurately diagnose and determine end points for clinical trials, or evaluate the usefulness of different medications in the treatment of dry eye disease. The recently developed fluorophotometer can objectively detect changes in the corneal epithelium by quantitatively measuring its barrier function or permeability. The purpose of the study is to investigate the use of corneal fluorescein penetration measured by the fluorophotometer as a diagnostic tool in the evaluation of dry eye patients.

**Methods:**

Dry eye patients (16 eyes), who presented with a chief complaint of ocular irritation corresponding with dry eye, low Schirmer's one test (<10 mm after 5 minutes) and corneal fluorescein staining score of more than two, were included in the study. Normal subjects (16 eyes), who came for refraction error evaluation, served as controls. Institutional Review Board (IRB) approved consent was obtained before enrolling the subjects in the study and all questions were answered while explaining the risks, benefits and alternatives. All Fluorophotometry of the central corneal epithelium was done utilizing the Fluorotron Master (TradeMark). Each eye had a baseline fluorescein scan performed, after which 50 l of 1% sodium fluorescein dye was instilled. Three minutes later, the fluorescein was washed with 50 ml of normal saline. Fluorescein scans were then started immediately after washing and were recorded at 10, 20, 40, and 60 minutes thereafter. The corneal peak values of fluorescein concentration were recorded within the central cornea in both dry eyes and in controls.

**Results:**

Ten minutes after fluorescein installition, patients with dry eye disease averaged a five-fold increase in corneal tissue fluorescein concentration (mean = 375.26 ± 202.67 ng/ml) compared with that of normal subjects (mean = 128.19 ± 85.84 ng/ml). Sixty minutes after dye installation, patients with dry eye disease still revealed higher corneal tissue fluorescein concentration (mean = 112.87 ± 52.83 ng/ml) compared with that of controls (mean = 40.64 ± 7.96 ng/ml), averaging a three-fold increase.

**Conclusion:**

Patients with dry eye disease demonstrated an increased corneal permeability and a slower rate of elimination to topically administered fluorescein when measured by the fluorophotometer. This suggests that fluorophotometry may serve as a valuable quantitative and objective tool for the diagnosis of dry eye disease, and in following patients' response to new treatment modalities. Fluorophotometry may serve as an objective non-invasive tool for end-point analysis in clinical trials of new treatments for dry eye disease.

## Background

The fluorophotometer can detect clinical and subclinical changes in the corneal epithelium by quantitatively measuring the barrier function of the corneal epithelium [1-3]. Corneal epithelial cells are associated with tight junctions that provide ocular protection by forming a resistant barrier to the passage of hydrophilic substances, macromolecules, and pathogens[7]. Several investigators have attempted to quantify this barrier function using fluorophotometry to measure the rate at which a topically applied ophthalmic dye, fluorescein, penetrates the cornea[8].

Fluorescein is a small, nontoxic, molecule that has the property of emitting light energy of a longer wavelength when stimulated by light of a shorter wavelength. The excitation peak for fluorescein molecules is about 490 nm (blue part of the spectrum) and represents the maximal absorption of light energy by fluorescein. Fluorescein molecules stimulated by this wavelength will be excited to a higher energy level and will emit light of a longer wavelength, which will be in the green portion of the spectrum at about 530 nm[9]. The fluorophotometry device Fluorotron Master™, (Ocumetrics, Mountain View, CA, USA) records the fluorescein concentration of different parts of the eye including the cornea and vitreous.

Fluorophotometry may be useful clinically because an increased corneal uptake of fluorescein reveals subtle damage to the corneal epithelium. In humans, measurements of the penetration of fluorescein across the corneal epithelium could be of value in diagnosing or monitoring dry eye disease. We were thus motivated in the present study to evaluate the corneal barrier function in dry eye patients to determine if fluorophotometry can be used as a clinical diagnostic test for dry eye disease.

## Methods

### Design and setting

This was a pilot assessment designed to investigate the reliability and consistency of the Fluorophotometer in diagnosing dry eye disease. Data was collected from dry eye patients to assess the permeability of fluorescein. This study was carried out in the department of ophthalmology at Mt. Sinai School of Medicine, New York. The research was designed as a case-control study. All patients examined received an informed consent, which was IRB approved.

### Subjects

IRB approved consent was obtained from all subjects prior to the screening procedure at the clinical research site. Informed consent was discussed with the patient, or surrogate, in detail. Every topic was addressed separately. All risks, benefits, and alternatives were discussed. Patient was allowed to ask questions, and all questions were answered. The assessment was made after that conversation, whether or not the patient understood the consent as described above. Subjects were then informed whether they were eligible to participate in the study after the screening was done.

Dry eye patients (16 eyes), who presented with a chief complaint of ocular irritation corresponding with dry eye, low Schirmer's one test without anesthesia (<5 mm after 5 minutes) and corneal fluorescein staining score of more than two, were included in the study. Normal subjects (16 eyes), who came for refraction error evaluation served as controls.

Fluorophotometry of the central corneal epithelium was done utilizing the Fluorotron Master™. Each eye had a baseline fluorescein scan performed, after which 50 μl of 1% sodium fluorescein dye was installed. Three minutes later, the fluorescein was washed with 50 ml of normal saline for sixty seconds. Fluorescein scans were then started immediately after washing and were recorded at 10, 20, 40, and 60 minutes thereafter. The corneal peak values of fluorescein concentration were recorded within the central cornea in both dry eyes and in controls. Each patient underwent a comprehensive ophthalmic examination including a review of medical history, best-corrected visual acuity, slit-lamp biomicroscopy, and dilated fundoscopic examination. Patients were excluded if they had a history of laser or ocular surgeries, and if they anticipate the use of any topical medication during the study period. Patients (16 eyes), who presented with a chief complaint of ocular irritation corresponding with dry eye, low Schirmer's one test (<10 mm after 5 minutes) and corneal fluorescein staining score of more than two, were included in the study.

After installation of fluorescein, the ocular surface was examined microscopically to evaluate the intensities of corneal staining (degree 0–3) and conjunctival staining (both nasally and temporally; degree 0–3). Fluorescein staining was scored as 0 (0), 1 (1–3), 2 (4–6) or 3 (>7). Those who had no symptoms of dry eyes and no other ocular problems other than refractive error served as controls (16 eyes).

### Demographics

Fluorophotometry was measured in 16 patients, which were classified as normal or dry eye patients according to their presenting primary ocular symptoms, their schirmer's test result and their corneal fluorescein staining score. A total of 32 eyes were analyzed. The mean age (± SD) of the dry eye patients and the controls was 47.50 (+ 17.86) and 37.17 (+13.49) years respectively. Among our patients, 9 were Caucasian, 3 were Asian, and 4 were of other ethnicity (Table 1).

**Table 1:** Patient Demographics. Fluorophotometry was measured in 16 patients, which were classified as normal or dry eye patients according to their presenting primary ocular symptoms, their schirmer's test result and their corneal Fluorescein staining score.

Number of Subjects in Our Study	16
Gender:	
Male	4
Female	12
Mean Age ± SD in Controls (years):	37.17 ± 13.49
Mean Age ± SD in Dry Eye Patients (years):	47.50 ± 17.86
Race:	
Caucasian	9
Asian	3
Others	4
Diagnosis:	
Normal Patients	8
Dry Eye Disease Patients	8

### Procedure

#### Fluorophotometry

Fluorophotometry of the central corneal epithelium was performed with the Fluorotron Master™ (Ocumetrics, Mountain View, CA) using the standard excitation and emission filters and the corresponding scanning software. This machine records the fluorescein concentration of different parts of the eye ranging from behind the crystalline lens to the space outside the cornea. Before any fluorescein was installed, a baseline scan, measuring each eye's intrinsic fluorescence, was performed using the Fluorotron Master. 50 μl of 1% sodium fluorescein dye (in the form of an eye drop) was then placed onto the tear film. After waiting for three minutes, the fluorescein was washed with 50 c.c. of normal saline. Fluorescein scans were recorded at 10, 20, 40, and 60 minutes after washing. The corneal peak values of fluorescein concentration were noted for each exam.

#### Data analysis

**Figure 1:**
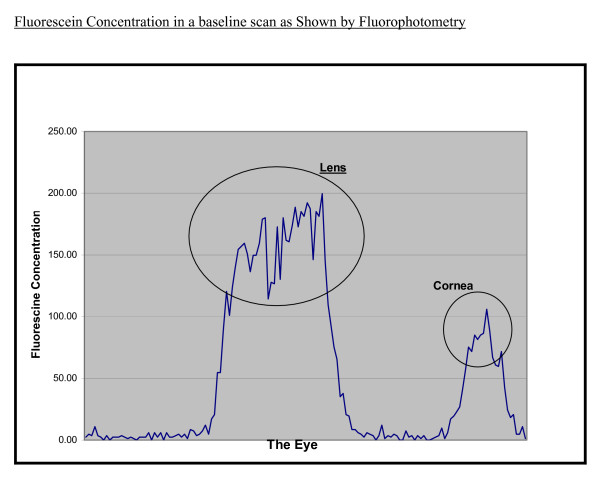
Patient Demographics: Subject view of the Fluotron results at baseline scan. The initial peaks indicate fluorescein concentration for the lens and the second peak indicates the fluorescein concentration in the cornea.

From the data obtained, graphs were created to display the fluorescein concentration (ng/ml) from behind the crystalline lens to the space outside the cornea for each time period scans (Figure 1). The corneal peak concentration values were recorded for analysis. The elimination rate of fluorescein from the eye was calculated by calculating the change in corneal fluorescein concentration over time. Statistical significance was determined utilizing the student t-test.

## Results

Ten minutes after fluorescein installation, patients with dry eye disease averaged a five-fold increase in corneal tissue fluorescein concentration (mean = 375.26 + 202.67 ng/ml) compared with that of normal subjects (mean = 128.19 + 85.84 ng/ml). Sixty minutes after dye installation, patients with dry eye disease still revealed higher corneal tissue fluorescein concentration (mean = 112.87 + 52.83 ng/ml) compared with that of controls (mean = 40.64 + 7.96 ng/ml), averaging a three-fold increase. The increase in corneal fluorescein concentration in dry eye patients is due to a defect in the corneal epithelial barrier function. Therefore, the stain tends to pool within the corneal epithelial layers for longer periods in patients with dry eye disease.

**Figure 2:**
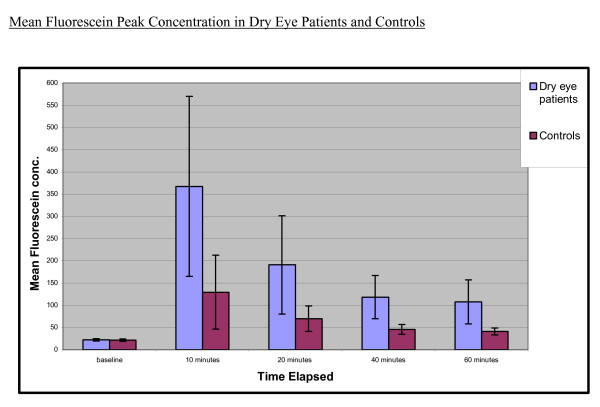
Corneal Peak Fluorescence: Demonstrates the comparison of Mean Fluorescein peak concentrations between dry eye patients and controls.

The baseline scans of the dry eye patient group (21.70 + 2.60 ng/ml) and control group (22.42 + 2.56 ng/ml) did not reveal a statistical difference. For each of the timed scans recorded after washing, there was a statistically significant difference (p < 0.05) in corneal fluorescein concentrations between the dry eye disease groups versus the control group (Table 2). The dry eye disease group had a higher corneal peak fluorescein concentration compared to the control group (Figure 2). A ratio of the corneal fluorescein concentration at 60 minutes to the baseline fluorescence was also used for comparison (Table 2). The dry eye disease group had a higher ratio than the control group, and the difference was statistically significant (p = 0.000277).

**Table 2:** Corneal Peak Fluorescence (ng/ml) at different times. Corneal Peak Fluorescence (ng/ml) was measured at different times. A ratio of the corneal fluorescein concentration at 60 minutes to the baseline fluorescence was also used for comparison.

		Baseline	10 minutes	20 minutes	40 minutes	60 minutes	60/baseline
DED	Mean	22.42	375.26	202.57	118.95	112.87	5.04
	St Dev	2.56	202.67	117.78	52.47	52.83	2.23
Normal	Mean	21.70	128.19	66.23	45.80	40.64	1.92
	St Dev	2.60	85.84	26.30	11.52	7.96	0.30
	P value	0.437675	0.00088774	0.001278239	0.00027737	0.0003374	0.000276759

* DED = Dry Eye Disease

We also estimated the elimination rate of fluorescein from the cornea between each of the scans. The elimination rate of fluorescein from the eye was statistically different when comparing the two groups (Table 3). It appeared that the rate of fluorescein elimination was greater in the dry eye group verse that of the control. Since the tear turnover rate is different between dry eye and normal controls, the elimination rate is of little significance.

**Table 3:** Elimination Rate of Fluorescein (ng/ml/min). Elimination Rate of Fluorescein (ng/ml/min) was recorded at various intervals. The elimination rate of fluorescein from the eye was statistically different when comparing the two groups.

		10–20 min rate	20–40 min rate	40–60 min rate	10–40 min rate
DED	Mean	17.27	4.18	0.30	8.54
	St Dev	11.71	4.08	0.34	5.41
Normal	Mean	6.20	1.02	0.26	2.75
	St Dev	7.28	1.21	0.40	2.87
	P value	0.008062675	0.017622827	0.7451916	0.002823332

* DED = Dry Eye Disease

## Discussion

Dry eye disease is a common source of great discomfort affecting people of all ages. It can seriously diminish a patient's quality of life, putting its greatest burden on the elderly population [11,12]. Its management can be a frustrating experience for patients and their eye care providers particularly because most clinicians still rely on traditional tests, such as tear breakup time, Schirmer, and ocular surface dye staining for diagnosis and follow up of dry eye disease. Many newer diagnostic tools aiming to improve sensitivity and specificity over conventional testing have emerged over the last several years.

These techniques include tear film osmolarity, scanning confocal microscopy, infrared thermography, fluorogenic substrate techniques and tear fluid protein immunoassays [4,5]. Although these techniques have not gained widespread clinical acceptance, the preliminary results indicate that tear composition and clearance appear to have a stronger correlation with the severity of ocular surface irritation symptoms in comparison to the Schirmer test, which evaluates tear production alone [11]. However these tests are difficult to utilize and are rarely used clinically and are in no way performed in clinical trials. Several studies report that the anterior fluorophotometer quantitatively measures the degree of breakdown of corneal epithelial barrier function [15-19]. Fluorophotometry may possibly be a new objective tool for diagnosing dry eye disease that is easy to operate.

Our results showed no statistical difference between the baselines scans. This was expected since the inherent fluorescence of the eye should theoretically be similar in both groups. However, the dry eye disease group had a significantly higher corneal peak fluorescein concentration than the control group at all scan times, suggesting an uptake of fluorescein in the superficial punctuate keratopathy. Based on this information we can measure the corneal fluorescence intensity and convert into fluorescein concentration. In aqueous-tear deficient dry eye patients fluorescein uptakes showed increased absorption of fluorescein from the tear film through the corneal epithelium i.e. greater severity in superficial punctuate keratopathy in dry eye patients.

There was a large standard deviation with a significant overlap in the levels of corneal fluorescein concentration and rate of fluorescein elimination in the early scans (at 10 and 20 min) between the dry eye patients and the controls. As the time from the original administration of the dye to the scan time increased, the amount of overlap between the data of the two groups decreased, thus improving the accuracy in distinguishing dry eye patients from the controls, and increasing the reliability of fluorophotometry as a diagnostic tool.

One possible explanation for the observed early variances in our data is the variation in the severity of dry eye disease in our patient group. Another is the different response of patients' eyes to fluorescein such as excessive tearing, blinking and eye squeezing. An additional reason is that the Fluorotron Master may have measured residual fluorescein in the tear film.

The early results are confounded by the high concentration in the tear film and the Fluorotron Master is unable to distinguish the tear film from the cornea, this would affect the measurements of peak corneal fluorescein concentration [12,13]. Previous studies have shown difficulties with calculating permeability where either corneal thickness [14] or tear film thickness [13] had to be assumed. Therefore, utilizing measurements at 60 minutes post fluorescein placement helped to more easily distinguish the two groups.

In conclusion, our data suggests that fluorophotometry may serve as a valuable quantitative and objective tool for the diagnosis of dry eye disease. In addition, peak corneal fluorescein concentration may generate a new classification system that would quantify mild, moderate and severe dry eye disease. Fluorophotometry may offer an objective non-invasive tool for endpoint analysis in clinical trials of new treatments for dry eye disease. Future evaluation of fluorophotometry and corneal permeability is warranted.

## Conclusion

Patients with dry eye disease demonstrated an increased corneal permeability and a slower rate of elimination to topically administered fluorescein when measured by the fluorophotometer. This suggests that fluorophotometry may serve as a valuable quantitative and objective tool for the diagnosis of dry eye disease, and in following patients' response to new treatment modalities. Fluorophotometry may serve as an objective non-invasive tool for end-point analysis in clinical trials of new treatments for dry eye disease.

## Abbreviations

DED – Dry Eye Disease

NEI – National Eye Institute

IRB – Institutional Review Board

## Competing interests

Commercial Relationships: Supported in part by NEI#EY01867, Research to Prevent Blindness, Inc. (to Asbell and department).

## Authors' contributions

MF has made substantial contributions to conception and design, or acquisition of data, or analysis and interpretation of data along with drafting the manuscript or revising it critically for important intellectual content. SH has made substantial contributions to conception and design, or acquisition of data, or analysis and interpretation of data along with drafting the manuscript or revising it critically for important intellectual content. CK has made substantial contributions to conception and design, or acquisition of data, or analysis and interpretation of data along with drafting the manuscript or revising it critically for important intellectual content. VF has made substantial contributions to conception and design, or acquisition of data, or analysis and interpretation of data along with drafting the manuscript or revising it critically for important intellectual content. PA has made substantial contributions to conception and design, or acquisition of data, or analysis and interpretation of data; drafting the manuscript or revising it critically for important intellectual content; and final approval of the version to be published.

## Pre-publication history

The pre-publication history for this paper can be accessed here:


